# Exploring Pelvic Symptom Dynamics in Relation to the Menstrual Cycle: Implications for Clinical Assessment and Management

**DOI:** 10.3390/jpm14030239

**Published:** 2024-02-23

**Authors:** Maria Blanco-Diaz, Ana Vielva-Gomez, Marina Legasa-Susperregui, Borja Perez-Dominguez, Esther M. Medrano-Sánchez, Esther Diaz-Mohedo

**Affiliations:** 1 Faculty of Medicine and Health Sciences, University of Oviedo, 33006 Oviedo, Spain; blancomaria@uniovi.es; 2 Physiotherapy and Translational Research Group (FINTRA), Institute of Health Research of the Principality of Asturias, 33003 Oviedo, Spain; 3 Department of Physiotherapy, Faculty of Health Sciences, University of Malaga, Campus Teatinos, 28078 Malaga, Spain; anavielva@uma.es (A.V.-G.); marinalegasa@uma.es (M.L.-S.);; 4 Exercise Intervention for Health Research Group (EXINH-RG), Department of Physiotherapy, University of Valencia, 46010 Valencia, Spain; francisco.b.perez@uv.es; 5 Department of Physiotherapy, University of Seville, Avenzoar St., 41009 Seville, Spain

**Keywords:** pelvic floor dysfunctions, menstrual cycle, menstruation, underdiagnosis, individualized medicine, education

## Abstract

Background: Pelvic floor dysfunctions (PFDs) encompass an array of conditions with discrepant classification systems, hampering accurate prevalence estimation. Despite potentially affecting up to 25% of women during their lifetime, many remain undiagnosed, underestimating the true extent. Objectives: This cross-sectional study aimed to examine the impacts of the menstrual cycle on PFDs and dysfunctions. Secondary objectives included investigating differences between athletic and nonathletic women. Methods: An online questionnaire examined the effects of the menstrual cycle (MC) on 477 women’s pelvic symptoms (aged 16–63 years), stratified by athletic status. This ad hoc instrument built upon a validated screening tool for female athletes. Results: Most participants reported symptom fluctuations across menstrual phases, with many modifying or reducing exercise participation. A concerning number experienced daily undiagnosed pelvic floor symptoms, emphasizing needs for comprehensive medical evaluation. Conclusions: Exacerbated pelvic symptoms showed complex relationships with menstruation, highlighting the importance of considering the MC in customized clinical management approaches. Symptoms demonstrated differential links to menstruation, indicating needs for individualized evaluation and tailored treatment plans based on symptom profiles and hormonal interactions. Educating professionals and patients remains essential to enhancing awareness, detection, and therapeutic outcomes. Further controlled longitudinal research should elucidate intricate relationships between menstrual cycles and pelvic symptom variability.

## 1. Introduction

Pelvic floor dysfunctions (PFDs) encompass a spectrum of conditions with varied definitions and classification schemes, leading to discrepancies in prevalence estimations [1].

Approximately a quarter of women may experience PFDs during their lifetime [2], with recent studies indicating prevalence rates of 32.0%, 5.5%, and 1.1% for one, two, and three categories of dysfunction, respectively [3]. Importantly, a substantial number of affected women may not seek healthcare, contributing to the underestimation of PFD prevalence [4].

Among PFD, urinary incontinence (UI) stands out, affecting 17.1% of women in the USA, with subcategories such as stress urinary incontinence (SUI), urgency urinary incontinence (UUI), and mixed urinary incontinence (MUI) [5]. Fecal incontinence (FI) affects 9.4% of women [2,6], while estimates for pelvic organ prolapse (POP) range from 3% to 8%, with variations based on symptom-based or examination-based classification [7]. Sexual dysfunctions (SDs) include hypoactive sexual desire disorder, arousal disorder, orgasmic disorder, and sexual pain disorder, with dyspareunia and vaginismus standing out in the pain disorder subgroup [8,9]. The prevalence of SD fluctuates between 30% and 50% in women [5], and chronic pelvic pain is reported in 14.8% of women over 25 years of age in the United Kingdom [10]. Surgical procedures for UI and POP have shown an increasing trend, with approximately 20% of women undergoing surgery for SUI and POP at some point in their lives, involving a high economic cost [2].

Risk factors for PFD include aging, menopause, history of hysterectomy, vaginal delivery, obesity, smoking, race, and genetic predisposition to connective tissue disorders [11]. Vaginal delivery is associated with a higher prevalence of POP, UI, and FI compared to cesarean delivery, while instrumentalized vaginal delivery is significantly associated with a higher risk of FI and POP [12].

The female reproductive system, a complex physiological system regulated by the menstrual cycle (MC), undergoes cyclic changes that influence various hormonal and regulatory components [13]. The MC, a monthly process lasting 21 to 35 days in healthy adult women, involves biological rhythms regulated by the hypothalamus–pituitary–ovary axis [14]. The MC comprises distinct phases—follicular (early and late subphases), ovulation, and luteal (early, middle, and late subphases)—governed by hormonal fluctuations. These phases orchestrate the maturation of reproductive cells, ovulation, and the formation and regression of the corpus luteum [15,16]. Notably, estrogen and progesterone, released by the ovaries in response to gonadotropins, play pivotal roles in reproductive function [15].

The MC’s influence on pelvic floor musculature, including alterations in perineal musculature activity, muscle tone, strength, and contraction drive, has been documented [17]. Given the reported rise in injuries among women compared to men and their potential association with menstruation, studies have explored links between the MC and injuries [18]. Variations in strength levels, hormonal fluctuations, including elevated estrogen levels associated with improved endurance and progesterone’s impact on cardiorespiratory effort during exercise, have been observed [19].

Given the complex nature of PFDs and their potential association with the MC, there is a compelling need for a comprehensive study to investigate this relationship.

The clinical significance of this study lies in its capacity to enhance personalized clinical management approaches for individuals grappling with pelvic floor symptoms. By elucidating the intricate relationships between the MC and PFDs, we strive for our findings to provide valuable insights that underscore the importance of comprehensive medical evaluations. This underscores the need for healthcare professionals and patients to be well informed about these issues, with the goal of optimizing detection, understanding, and therapeutic management. Consequently, this study not only contributes valuable insights into the dynamics of pelvic disorders but also advocates for a paradigm shift towards personalized and informed clinical practices. This underscores the necessity of considering the MC in both diagnostic and therapeutic processes, highlighting the nuanced complexity of pelvic symptoms and endorsing the adoption of individualized treatment plans.

This study aimed to examine the effect of the MC on symptoms and/or dysfunctions of the pelvic floor, as well as to observe potential differences between women who engage in sports and those who do not.

## 2. Materials and Methods

### 2.1. Study Design 

This cross-sectional study involved voluntary participation, ensuring that respondents remained anonymous, and no financial incentives were provided. Prior to completing the questionnaire, informed consent was provided by the participants. 

The inclusion criteria for participants were women between 16 and 63 years of age with or without previously diagnosed pelvic floor pathologies, informed consent, and voluntary participation in this study. Exclusion criteria were menopausal participants and those unable to complete the questionnaire.

The study addressed demographic variables, assessing participants’ age, age at menarche, and parity. Occupational variables encompassed a diverse range of professions, including healthcare workers, students, individuals in business, education, engineering, and arts. Various pelvic symptoms were explored, spanning urinary incontinence, gas incontinence, fecal incontinence, urinary urgency, pelvic pain, vaginal heaviness sensation, dyspareunia, diarrhea, and constipation. The study emphasized the prevalence of daily pelvic symptoms, and it delved into modifications in sports habits during the MC, highlighting variations in symptom patterns across different menstrual phases [20].

We also assessed study quality using the Strengthening the Reporting of Observational Studies in Epidemiology (STROBE) checklist (Supplementary Table S1) [21].

### 2.2. Participants

The recruitment of volunteers took place through social media and in-person appeals at both private clinics and public hospitals within the national healthcare network. A sample size of 385 participants was calculated based on an α of 0.05, a standard error of 5%, and a confidence interval of 95%.

The final sample consisted of 477 women who volunteered for the study.

### 2.3. Data Collection

Data were collected through 594 online questionnaires administered from April to October 2023. Eligible participants were provided with a general description of the study, information about what participation entailed, and were invited to provide their informed consent.

Participants were assured that their personal information would remain confidential and not be disclosed to any third party or institution, in compliance with the applicable laws.

Upon agreeing to participate in the study, they were granted access to the questionnaire.

### 2.4. Data Analysis

The statistical software R version 4.3 was used for data analysis. Sociodemographic data are presented as frequencies and percentages. 

Linear and logistic regression models were utilized to generate odds ratios (ORs), alongside multiple regression models. Additionally, Chi-square and Fisher’s exact tests were performed on frequency tables, adjusting the analysis based on the number of cases in each category.

A statistically significant difference was accepted at a *p*-value < 0.05.

### 2.5. Instruments

An ad hoc questionnaire based on the validated LEAF-Q questionnaire for detecting early female athletes at risk of Relative Energy Deficiency in Sport (RED-S) was utilized [20]. The questionnaire consisted of 27 items categorized into 3 sections: (i) personal data (4 items), (ii) MC and pelvic–perineal health information (17 items), and (iii) details regarding sporting activity (5 items). A final voluntary section was also included for those who wished to add any additional comments.

### 2.6. Ethical Considerations

The research adhered to the principles of the Declaration of Helsinki, and ethical research guidelines were strictly followed. Approval for the study protocol was obtained from the Ethics Committee of the University of Malaga, emphasizing the integrity and ethical standards maintained throughout the research process.

## 3. Results

A total of 477 women were included in the study, with an average age of 30.69 years (SD ± 8.82). The mean age at menarche was 12.5 years (SD ± 1.55). Among the participants, 10.1% had one childbirth, 8.8% had two childbirths, and 2.1% had three or more childbirths. In terms of occupation, 28.9% of the participants were employed in healthcare, 15.9% were students, 14.9% worked in business, 13.6% were in education, 2.7% were engineers, and 1.7% identified as artists. 

Regarding symptoms during daily activities, 42.6% of the participants experienced them, 41.9% experienced symptoms at rest, and 18.25% experienced symptoms during sports activities. Regarding modifications in sports habits during the MC, 63.1% of women were compelled to adjust their exercise or its intensity during the MC, 25.4% never modified it, 10.8% could not engage in sports due to symptoms during menstruation, and 0.7% reported other reasons.

Furthermore, the questionnaire demonstrated satisfactory internal consistency, as evidenced by a Cronbach’s Alpha value of 0.8141.

Concerning the daily symptoms reported by the participants and their correlation with medical diagnoses, several relationships emerged. Among the participants, 68.6% had at least one pelvic symptom, with 74.6% undiagnosed and 25.4% diagnosed. Of the 2.7% who experienced FI symptoms, 61.5% lacked a diagnosis. Similarly, 54.8% of the 8.8% of participants with gas incontinence were undiagnosed. Among the participants, 24.9% had daily diarrhea, with 71.4% being undiagnosed. For the 20.8% of participants with constipation, 63.6% were undiagnosed compared to 36.4% who were diagnosed. Urge incontinence occurred in 13.2% of participants, with 58.7% undiagnosed. Additionally, 41.3% of participants experienced pelvic pain, which was diagnosed in 26.9% and undiagnosed in 73.1%. Regarding dyspareunia, 23.3% of participants reported this, which was diagnosed in 56.8% and undiagnosed in 43.2%. For UI, 13.2% of participants were affected, with 41.7% diagnosed and 58.3% undiagnosed. The median underdiagnosis rate for daily pelvic symptoms was 67.6% (Table 1).

**Figure 1:**
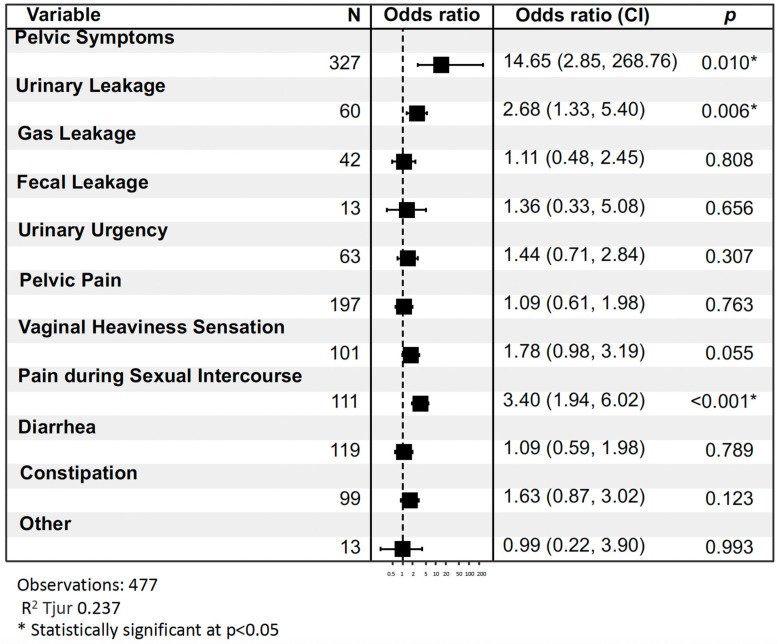
Association of pelvic symptoms with pelvic dysfunction: odds ratios and confidence intervals.

The following statistically significant results were identified: Having at least one pelvic symptom increases the likelihood of having pelvic dysfunction by 14.65 times compared to having none (OR = 14.65, 95% CI = [2.85, 268.76], *p*-value = 0.010). However, it is important to note that the confidence interval is wide, indicating substantial variability. Experiencing UI increases the likelihood of pelvic dysfunction by 2.68 times compared to not experiencing it (OR = 2.68, 95% CI = [1.33, 5.40], *p*-value = 0.006). Similarly, experiencing dyspareunia increases the likelihood of pelvic dysfunction by 3.40 times compared to not having it (OR = 3.40, 95% CI = [1.94, 6.02], *p*-value < 0.001) (Figure 1).

The analysis of symptom patterns in relation to the MC revealed a diverse landscape among the participants. A notable portion reported distinct experiences of symptom occurrence or escalation across different menstrual phases. The majority, comprising 41.3% of participants, reported heightened symptoms during the initial days of their menstrual period. Preceding menstruation, 38.8% experienced the onset or intensification of symptoms. A smaller subset, 12.2% of participants, reported consistent symptom experiences throughout their entire MC, while 7.3% mentioned no noticeable increase in symptoms correlated with menstruation.

Furthermore, a minority experienced symptom exacerbation during specific menstrual phases: 5.5% during the middle days and 4.0% towards the end of menstruation.

**Figure 2:**
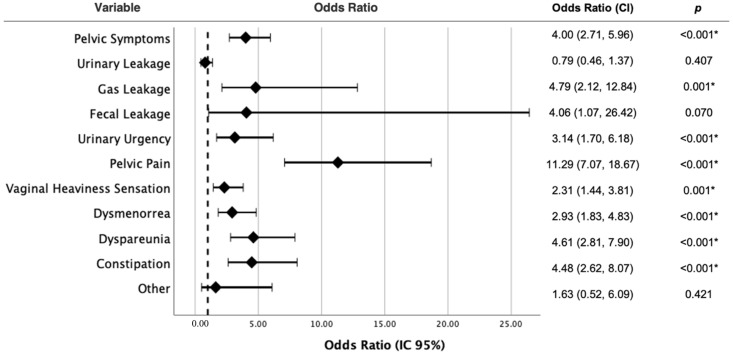
Relationship between having at least one pelvic symptom versus whether symptoms increase with menstruation. * Statistically significant at *p* < 0.05.

The following analysis investigated the relationship between experiencing at least one pelvic symptom, and each of them individually, in relation to an escalation of these symptoms during menstruation. Statistically significant results revealed that an escalation of symptoms during menstruation increases the likelihood of having any pelvic symptom by a factor of 4.00 compared to not experiencing such an escalation (OR = 4.00, 95% CI = [2.71, 5.96], *p*-value < 0.001). Similarly, an escalation in symptoms during menstruation raises the risk of experiencing gas incontinence by a factor of 4.79 compared to not having such an escalation (OR = 4.79, 95% CI = [2.12, 12.84], *p*-value = 0.001). Furthermore, an increase in symptoms during menstruation multiplies the risk of urinary urgency in daily life by 3.14 (OR = 3.14, 95% CI = [1.70, 6.18], *p*-value < 0.001). Likewise, an escalation of symptoms during menstruation elevates the risk of pelvic pain by 11.29 compared to not having such an escalation (OR = 11.29, 95% CI = [7.07, 18.67], *p*-value < 0.001). Similarly, experiencing an increase in symptoms during menstruation multiplies the risk of vaginal heaviness sensation by 2.31 (OR = 2.31, 95% CI = [1.44, 3.81], *p*-value = 0.001). Analogously, an escalation of symptoms during menstruation increases the risk of pain during sexual relations by 2.93 (OR = 2.93, 95% CI = [1.83, 4.83], *p*-value < 0.001). Additionally, an escalation of symptoms during menstruation raises the risk of diarrhea by 4.61 (OR = 4.61, 95% CI = [2.81, 7.90], *p*-value < 0.001). Finally, experiencing an increase in symptoms during menstruation multiplies the risk of constipation by 4.48 compared to not having such an escalation (OR = 4.48, 95% CI = [2.62, 8.07], *p*-value < 0.001) (Figure 2).

## 4. Discussion

The study sample consisted of 477 women, with an average age of 30.69 years (SD ± 8.82), including women of different age groups from 16 to 63 years. The reported age at menarche in this study’s sample was 12.5 years (SD ± 1.55). Previous research on European populations indicates that the normal age range for a girl’s first menstrual period falls approximately between 12 and 13 years [22,23]. Therefore, the mean age of menarche observed among the participants in this study is aligned with typical developmental patterns and the expected onset of menstruation. Regarding parity, most participants had one or two children, or none. Only a small percentage had three or more children (2.1%).

The predominant occupations among participants were healthcare workers, students, individuals in business, and those in education. This might partly relate to the recruitment methods and centers where the study was conducted. However, having a diverse sample in terms of occupations allows for exploring potential outcome differences between various employment groups.

The age range within the sample allows us to analyze pelvic symptoms in women. Previous studies have also focused on this population of young and adult women to investigate disorders like UI, pelvic pain, sexual dysfunctions, and other pelvic floor problems.

A significant number of participants reported substantial variations in the occurrence and intensity of symptoms across various menstrual phases. The majority highlighted an escalation of symptoms during the onset of their MC (80.1%), consistent with findings from other published studies [24].

A wide variety of physical-activity-related symptoms was observed among the participants. Notably, 42.6% of participants reported experiencing symptoms during daily activities, indicating a significant impact. Furthermore, over half of them (63.18%) stated the need to modify or reduce their exercise due to said symptoms. Especially relevant is that 10.8% were unable to perform any physical activity during menstruation due to these symptoms. This underscores the substantial impact on regular sports participation during this period. Authors like Martínez-Fortuny, N. et al. observed that hormonal fluctuations throughout the MC alter variables such as laxity, strength, body temperature, and neuromuscular control, among others. This means that women are constantly adapting to hormonal variations, exposing them to a higher risk of injuries [18].

In summary, the data indicate that MC-associated symptoms affect the physical activity of a broad percentage of evaluated women in highly varied ways. From causing discomfort in everyday tasks to preventing sports practice in severe cases, the consequences spectrum appears to be extensive. This emphasizes the need for strategies to manage these symptoms while mitigating their repercussion on women’s quality of life.

A staggering 68.6% of participants reported at least one pelvic symptom, significantly impacting their daily lives. The alarming proportion of women reporting daily pelvic symptoms without a medical diagnosis (74.6%) is concerning. This discrepancy between the high prevalence of symptomatic cases and low diagnostic confirmation suggests a potential under-reporting and under-recognition of these conditions among the female population, data similar to those collected by other authors [25]. The psycho-social barriers associated with sensitive intimate health matters emphasize the necessity for creating confidential, nonjudgmental spaces to facilitate timely detection. It is crucial to acknowledge that reluctance to discuss or embarrassment when discussing pelvic issues might act as a barrier, potentially hindering individuals from seeking appropriate healthcare support for these symptoms [26].

The high proportion of symptomatic participants without a diagnosis highlights the critical need for timely and accurate diagnosis procedures. The lack of diagnosis in many participants likely reflects challenges recognizing and understanding pelvic dysfunction symptoms [4]. This is especially relevant for symptoms like FI, gas incontinence, diarrhea, constipation, and urinary urgency, as lack of diagnosis can result in inadequate management and reduced quality of life. For instance, a striking 61.5% lacked a diagnosis despite experiencing FI symptoms, emphasizing a critical gap in their medical evaluation. Similarly, over half of the participants experiencing gas incontinence (54.8%) lacked a formal diagnosis. The high prevalence of diarrhea and constipation, with a significant portion remaining undiagnosed (71.4% and 63.6%, respectively), underscores the potential under-recognition of gastrointestinal-related pelvic symptoms in clinical practice, coinciding with the data observed by Meyer et al. [27]. Moreover, in cases of dyspareunia and UI, a considerable number of participants remained undiagnosed, despite the adverse impact on their quality of life; these data are consistent with the ACOG Practice Bulletin Clinical Management Guidelines for Obstetricians and Gynecologists, indicating that although female sexual dysfunction is relatively prevalent, women are unlikely to discuss it with their healthcare providers unless asked [28], and these data align with the findings from other researchers, indicating that a growing population of people experience underestimated lower urinary tract symptoms [29].

The subjective nature of these symptoms and potential lack of awareness among healthcare professionals regarding the need for comprehensive evaluations may contribute to this scenario. These findings underscore the need for education campaigns targeting both healthcare professionals and women with pelvic dysfunction symptoms, their impacts on quality of life, and the crucial significance of proper evaluation and management.

Diagnostic approaches require optimization; only then can the true magnitude of the impact from these conditions be ascertained and the necessary comprehensive care be provided.

Analysis concerning the MC revealed that a significant number of participants reported increased symptoms in the days leading up to and during menstruation. These findings emphasized the relevance of the early menstrual phase in relation to symptom exacerbation. Several authors have compared exercise performance between the early follicular phase and a combined assessment of all other phases (late follicular, ovulation, early luteal, middle luteal, and late luteal). Their findings suggest a potential minor decrease in exercise performance during the early follicular phase of the MC when compared to the collective performance across all other phases [23,30]. These variations in symptom patterns underscore the significance of recognizing individual differences when assessing and treating pelvic dysfunction symptoms in women. It underscores the need to employ individualized approaches that evaluate the dynamics of each woman’s symptoms in relation to her MC.

The results highlight the differential influence of various pelvic symptoms on exacerbation during menstruation. However, not all symptoms analyzed in this study seem to be equally affected by the MC, exhibiting varying degrees of influence. Some presented a clear association and tendency toward worsening symptomatology linked to menstruation, while in others, this relationship was not observed as clearly. Therefore, these findings underscore the importance of considering the specific nature and impact of each type of pelvic symptom in the clinical management and treatment strategy for said conditions. A careful analysis of how they manifest and exacerbate in the context of the MC could optimize the diagnosis, control, and therapy of pelvic problems in women, especially those that show a closer interaction with hormonal variation or other factors associated with menstruation. Considering these nuanced differences in symptom presentation could facilitate improved decisions for comprehensive management. Gynecological diseases are heavily associated with dysregulated steroid hormones and can induce chronic pelvic pain, dysmenorrhea (menstrual cramps), dyspareunia, heavy bleeding, and infertility, which substantially impact the quality of women’s lives. Because the MC repeatably occurs during reproductive ages with dynamic changes and remodeling of reproductive-related tissues, these alterations can accumulate and induce chronic and recurrent conditions [31].

Among all the symptoms documented in this study, patients experiencing pelvic pain exhibit the highest likelihood of increased pain during menstruation. Hellman et al. propose that cramping pain might result from a combination of uterine pressure and hemodynamic dysfunction [32]. This could be associated with pelvic congestion syndrome (PCS), a common cause of chronic pelvic pain in women of reproductive age or to the existence of chronic pelvic pain itself. Notably, approximately 10–20% of gynecological consultations are due to complaints of chronic pelvic pain, and only 40% of them are referred for evaluation by a specialist [33]. Furthermore, the participants in this study present endometriosis as the second symptom worsening with menstruation, which coincides with previous research, such as the study by Vercellini et al. in which they showed that women with endometriosis had a significantly higher rate of abnormal menstrual scores (dysmenorrhea, flow duration, cycle length) than those without the disease [34]. Similarly, Sachedina A. et al. noted that endometriosis worsens with continued menstruation and advised patients on menstrual suppression until pregnancy is desired [22]. Patients affected by endometriosis may commonly exhibit classic symptoms of dysmenorrhea (80%), dyspareunia (30%), dysuria, and dyschezia [35]. This evaluation partly elucidates why, in this study, everyday pelvic pain and endometriosis were significantly linked to increased symptoms during menstruation.

The significant relationship found between increased gastrointestinal (GI) symptoms such as diarrhea, constipation, and flatulence during menstruation is consistent with research exploring the influence of hormones on gastrointestinal motility. A growing number of clinical and experimental evidence strongly supports a crucial role of sex hormones in the regulatory mechanisms of the brain–gut–microbiota axis [36]. However, during the days leading up to the onset of natural cycle menstruation, estrogen and progesterone are produced by the ovaries in preparation for a possible pregnancy. At menstruation, there is a sudden drop in these hormones and a simultaneous spike in prostaglandins, which contract local smooth muscle tissue [24,37].

The lack of a statistically significant association between increased symptoms such as UI or POP during menstruation aligns with previous research, such as the work of Hvidman and Lone et al. (2002) who suggested that there is limited covariation in the occurrence of UI with the natural phases of the MC, which could explain the proximity but lack of statistical significance in this study [38]. Likewise, Connell et al. also did not find a clear association between the MC and POP, establishing that the relationship between genes and uterine prolapse is not fully established, but research is underway to better understand the potential connection. How genes expression and function relate to the hormonal and structural changes in the MC could provide crucial information on the underlying mechanisms of uterine prolapses [39]. However, these findings are not consistent with those of authors who have identified a relationship between UI and hormonal fluctuations that occur during the MC, due to the estrogen and progesterone receptors found in the female lower urinary tract [40,41]. Estrogen receptors are present in various areas, including the squamous epithelium of the proximal and distal urethra, vagina, bladder trigone, pubococcygeus, and pelvic floor muscles, excluding the levator ani. Changes in muscle function in these regions could potentially contribute to UI [40].

It is important to highlight that identifying these associations provides a solid foundation for improving the clinical management of pelvic symptoms. However, controlled studies are required to better understand the precise dynamics between pelvic symptoms and the MC, as well as to discern the direct influence of hormones on these symptoms.

In summary, this study significantly contributes to the field by highlighting the relationship between pelvic symptoms and their exacerbation during menstruation. These findings underscore the need for comprehensive approaches in managing pelvic symptoms that carefully consider the influence of the MC in order to enhance quality of life and treatment for these conditions.

### Limitations

Selection bias may exist when recruiting participants from specific clinics and social networks, which restricts demographic representativeness. Information bias may occur when using self-reported questionnaires, affecting the accuracy of the data. Confounding factors such as medical history were not controlled for, allowing them to influence the results. The lack of confirmed diagnoses indicates risks of reporting bias, potentially misrepresenting the prevalence of the disorder. Additionally, the questionnaire utilized in this study was developed ad hoc based on the LEAF-Q questionnaire for detecting early female athletes at risk of Relative Energy Deficiency in Sport (RED-S), a validated tool. However, the ad hoc instrument itself has not undergone formal validation, introducing a potential source of bias. Finally, the variability in respondents’ comfort with disclosing sensitive health details poses a risks of response bias.

## 5. Conclusions

A significant association was observed between the exacerbation of various pelvic symptoms and the early menstrual phase, emphasizing the importance of considering the MC in their clinical management.

Not all analyzed symptoms showed a clear link with menstruation, highlighting the necessity for individualized approaches depending on the type of symptom and its interaction with hormonal factors.

The high prevalence of pelvic symptoms with the absence of medical diagnosis emphasizes the need for a comprehensive evaluation of these potentially underrated, subjective symptoms.

Educational strategies targeting healthcare professionals and patients are required to optimize the detection, understanding, and therapeutic management of pelvic disorders given their impact on quality of life.

Although some initial associations were established, further longitudinal controlled studies are warranted to elucidate precise relationships between the MC and pelvic symptoms.

## Figures and Tables

**Table 1:** Daily symptoms reported and their correlation with pelvic dysfunction diagnosed.

	Pelvic Dysfunction Diagnosed					
Daily Pelvic Symptoms	Total (n)	No (n)	Yes (n)	Total (%)	No (%)	Yes (%)
Pelvic Symptoms	327	244	83	68.6%	74.6%	25.4%
Urinary Incontinence	60	35	25	12.6%	58.3%	41.7%
Gas Incontinence	42	23	19	8.8%	54.8%	45.2%
Fecal Incontinence	13	8	5	2.7%	61.5%	38.5%
Urinary Urgency	63	37	26	13.2%	58.7%	41.3%
Pelvic Pain	197	144	53	41.3%	73.1%	26.9%
Vaginal Heaviness Sensation	101	63	38	21.2%	62.4%	37.6%
Dyspareunia	111	63	48	23.3%	56.8%	43.2%
Diarrhea	119	85	34	24.9%	71.4%	28.6%
Constipation	99	63	36	20.8%	63.6%	36.4%
Other	13	9	4	2.7%	69.2%	30.8%
	1.145	774	371	100.0%	67.6%	32.4%

## Data Availability

Data are contained within the article and Supplementary Materials.

## Supplementary Materials

The following supporting information can be downloaded at: https://www.mdpi.com/article/10.3390/jpm14030239/s1, Table S1: Strobe statement.
